# Emerging role of immunoproteasomes in pathophysiology

**DOI:** 10.1038/icb.2016.50

**Published:** 2016-06-14

**Authors:** Gagandeep Kaur, Sanjay Batra

**Affiliations:** Environmental Toxicology PhD program, 207 Health Research Center, Southern University and A&M College, Baton Rouge, LA, USA

## Abstract

The immunoproteasome is a proteasome variant that is found only in jawed vertebrates. It is responsible for degrading intracellular proteins to generate a major source of peptides with substantial major histocompatibility complex I binding affinity. The immunoproteasome also has roles in T-cell survival, differentiation and proliferation in various pathological conditions. In humans, any alteration in the expression, assembly or function of the immunoproteasome can lead to cancer, autoimmune disorders or inflammatory diseases. Although the roles of the immunoproteasome in cancer and neurodegenerative disorders have been extensively studied, its significance in other disease conditions has only recently become known. Therefore, there is renewed interest in the development of drugs, vaccines and biomarkers that target the immunoproteasome. The current review highlights the involvement of this complex in disease pathology in addition to the advances made in immunoproteasome research.

## INTRODUCTION

Nearly 2 × 10^6^ proteins are degraded per minute in a cell. The ubiquitin proteasome system is the cellular machinery that makes this mammoth task possible.^1^ A proteasome is a barrel-shaped structure composed of a 20S core made up of four heptameric rings with 19S caps on either end of the core. This system functions to recognize and degrade misfolded, damaged or abnormal proteins that have been ubiquitylated and thus marked for degradation. The 19S cap recognizes these ubiquitylated proteins and directs them into its 20S core where they are degraded into smaller peptides.^2^ There are three tissue-specific proteasome variants in eukaryotes: the thymoproteasome (which is constitutively found in thymic epithelial cells and is responsible for the positive selection of developing T cells), the spermatoproteasome (which is constitutively found in the testes and is required for spermatogenesis) and the immunoproteasome (which is induced during immune responses in all jawed eukaryotes).^3^

The immunoproteasome is an inducible proteasome that is produced in most cells of the body in response to cytokines (especially interferon-γ (IFNγ)). A well-defined function of immunoproteasomes is the production of pools of peptides, some of which escape degradation in the cytosol and are loaded on major histocompatibility complex (MHC)-I for antigen presentation, leading to elicitation of the T cell-mediated immune response.^4^ However, this function is shared between immuno- and standard proteasomes which adds to the complexity of the immune response.^5^ Immunoproteasomes have also been closely linked to the emergence of various autoimmune disorders, inflammatory diseases and cancers in humans,^6^ thus making them a potential therapeutic target for various pathological conditions. Indeed, the proteasome inhibitor bortezomib has long been on the market as an Food and Drug Administration (FDA)-approved therapy for multiple myelomas.^7^ Many other proteasome inhibitors are being tested for their efficacies in the treatment of various types of cancer. However, these inhibitors indiscriminately target both constitutive proteasomes and immunoproteasomes, which results in various unwanted side effects. To overcome these ill-effects, efforts are being made to design selective inhibitors that specifically target immunoproteasome subunits.^6^ From this perspective, the potential for the utilization of immunoproteasome inhibitors in the treatment of diseases is worth exploring.^8,9^ Therefore, this review explores the relationship between the immunoproteasome profiles and various pathological conditions to guide future research.

## IMMUNOPROTEASOMES: STRUCTURE, ASSEMBLY, REGULATION AND FUNCTION

### Structure

**Figure 1:**
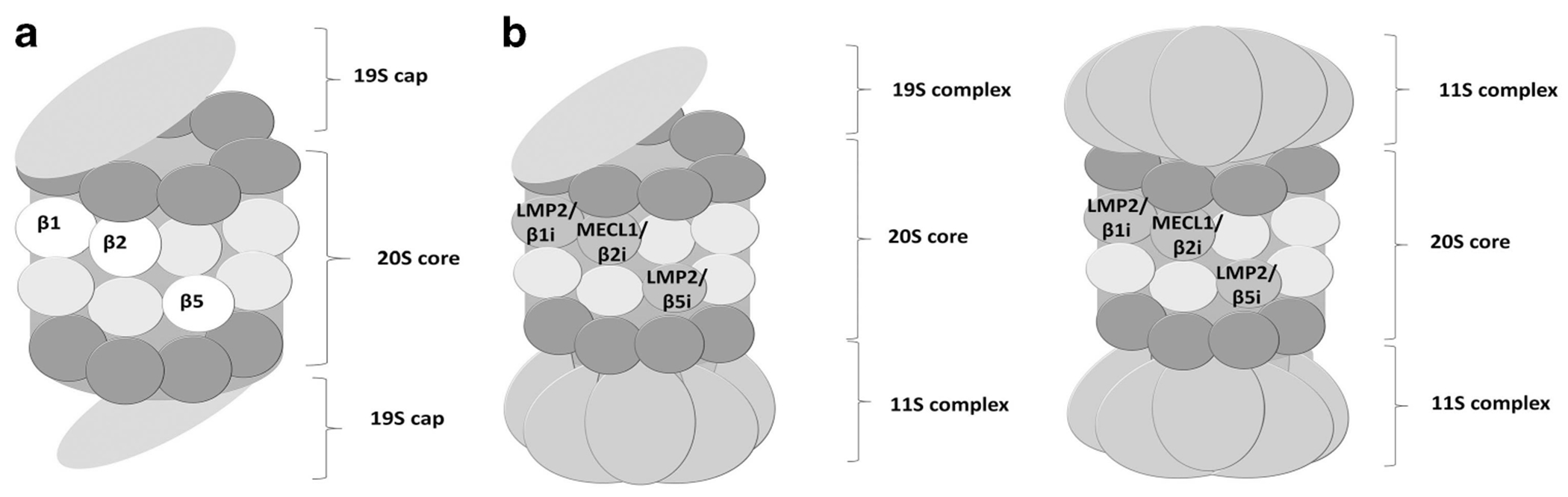
Structures of constitutive (**a**) and immunoproteasomes (**b**). The three subunits found in constitutive proteasomes (β1, β2 and β5) are replaced by inducible subunits (β1i, β2i and β5i) in immunoproteasomes. In addition, immunoproteasomes can contain two regulatory complexes, 19S and 11S, as shown above. A full color version of this figure is available online at the *Immunology and Cell Biology* website.

Although immunoproteasomes share 60–70% sequence homology with standard catalytic proteasomes (Figure 1a),^5^ they differ from constitutive proteasomes in several respects (Table 1). Structurally, an immunoproteasome is a multi-catalytic and multi-subunit protease composed of 28 subunits that form the 20S barrel-like core as determined based on the crystallographic structure in yeast (Figure 1b). This core is assembled into four stacked heptameric rings arranged as αββα. The only structural difference between standard proteasomes and immunoproteasomes lies in the β-subunits. The outer rings of both of these complexes are formed by non-catalytic α-type subunits, and the inner rings are composed of β-type subunits, three of which have catalytic enzyme activities. Although the β-subunits of standard proteasomes are called β1 (PSMB6), β2 (PSMB7) and β5 (PSMB5), the IFN-γ-inducible β subunits are named β1i/low-molecular-weight protein 2 (LMP2), β2i/multi-catalytic endopeptidase complex like-1 (MECL1) and β5i/low-molecular-weight protein 7 (LMP7).

These subunits have caspase-like (LMP2 subunit), trypsin-like (MECL1 subunit) and chymotrypsin-like (LMP7 subunit) activities, and cleave peptide bonds following acidic, basic and hydrophobic amino acid residues, respectively.^2^ The three inducible subunits contain removable pre-sequences and are homologous to a constitutive subunit, which enables them to serve as replacements for their homologs during proteasome assembly.^10^ The centers of the α-rings aid in the prevention of substrate proteins from penetrating the inner cavity where the β-ring and the proteolytically active sites are located. Thus, it is possible for a substrate to access the active site only by passing through the narrow opening that is found at the center of the α-ring.^11^

**Figure 2:**
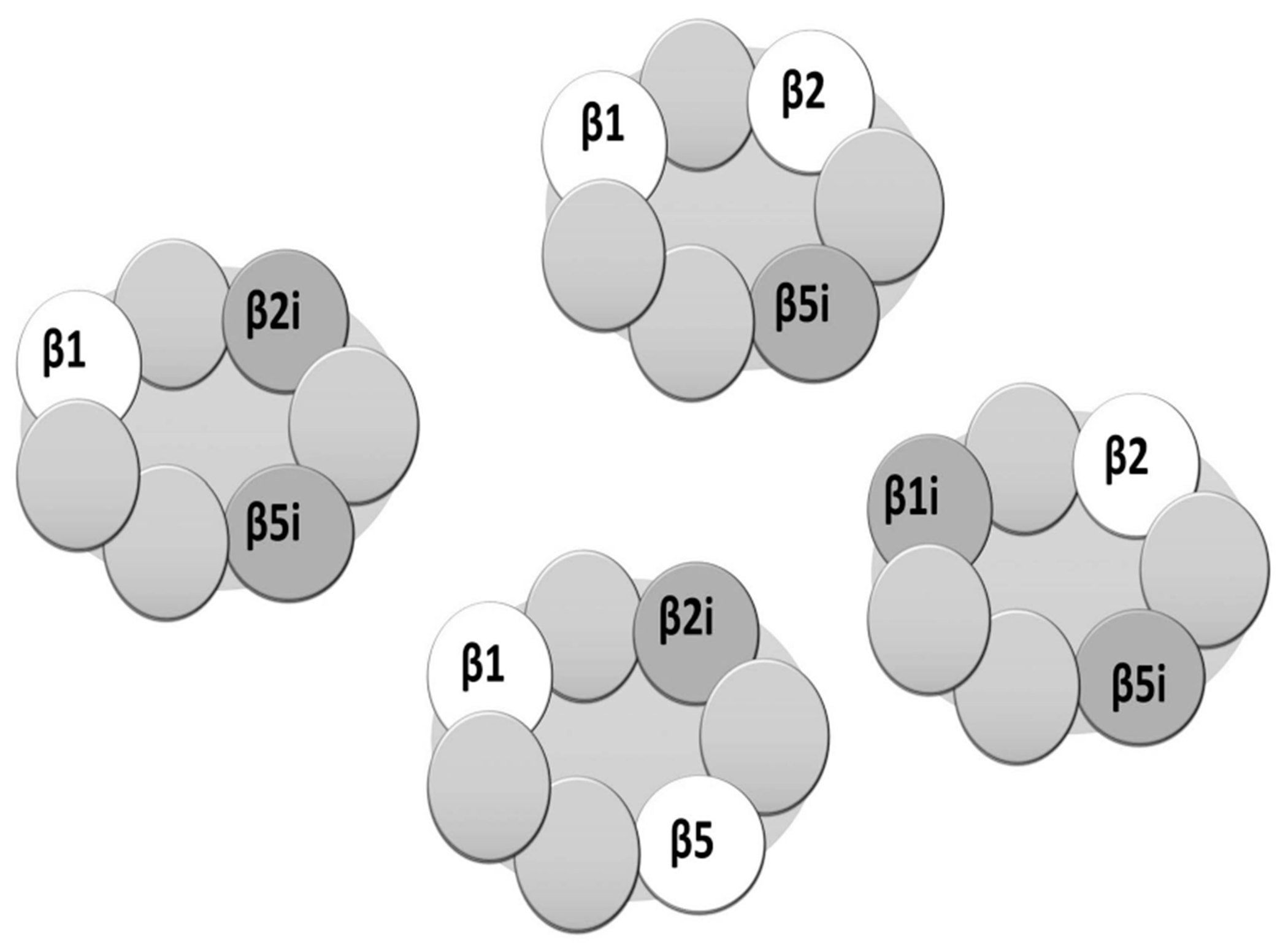
Schematic representation of the subunit arrangements that compose mixed proteasomes. Mixed proteasomes are composed of both constitutive and inducible subunits; thus, it is possible for numerous combinations of mixed proteasomes to be present in organisms. However, elaboration of their functions is beyond the scope of this review. A full color version of this figure is available online at the *Immunology and Cell Biology* website.

The regulatory particles PA28 (also known as 11S) and PA700 (also called 19S) serve as the closing gates for the core particles at the opening of the α-ring and act as proteasome activators to form the 26S immuno-proteolytic machinery. Specifically, these particles recognize polyubiquitylated proteins, translocate these proteins into the interior of the central core and deubiquitylate polyubiquitylated proteins to recycle the moieties.^12^ Under certain circumstances, the 19S complex is replaced by the 11S complex, and the associated advantage is that the 11S complex is much smaller and more efficient at generating antigenic peptides for presentation.^13^ The 19S/11S regulatory subunits function to direct the protein molecules into the 20S core, which in turn is responsible for ATP-dependent (in the case of the 19S subunit) or ATP-independent (in the case of the 11S subunit) degradation of antigenic proteins for presentation by MHC class I molecules.^5^ In general, immunoproteasomes are prevalent in cells of hematological origin, predominantly lymphocytes and monocytes; however, following induction by cytokines, including IFN-γ and TNF-α, immunoproteasomes are also expressed in non-hematologic cells. In addition, population of hybrid variants composed of inducible immunoproteasome subunits complexed with standard proteasome subunits have been found in mouse heart tissues and in human tumor, colon, liver, small intestine, kidney and dendritic cells. These variants are known as ‘mixed proteasomes’ (Figure 2). It is believed that these variants display unique abilities of antigen processing and thereby expand the pool of antigens presented by specific cells.^14,15^ Nonetheless, there seems to be a major crossover between the functions of standard, mixed and immunoproteasomes, and additional work is needed to elucidate the significance of each of these types of proteasome variants.

### Assembly

**Figure 3:**
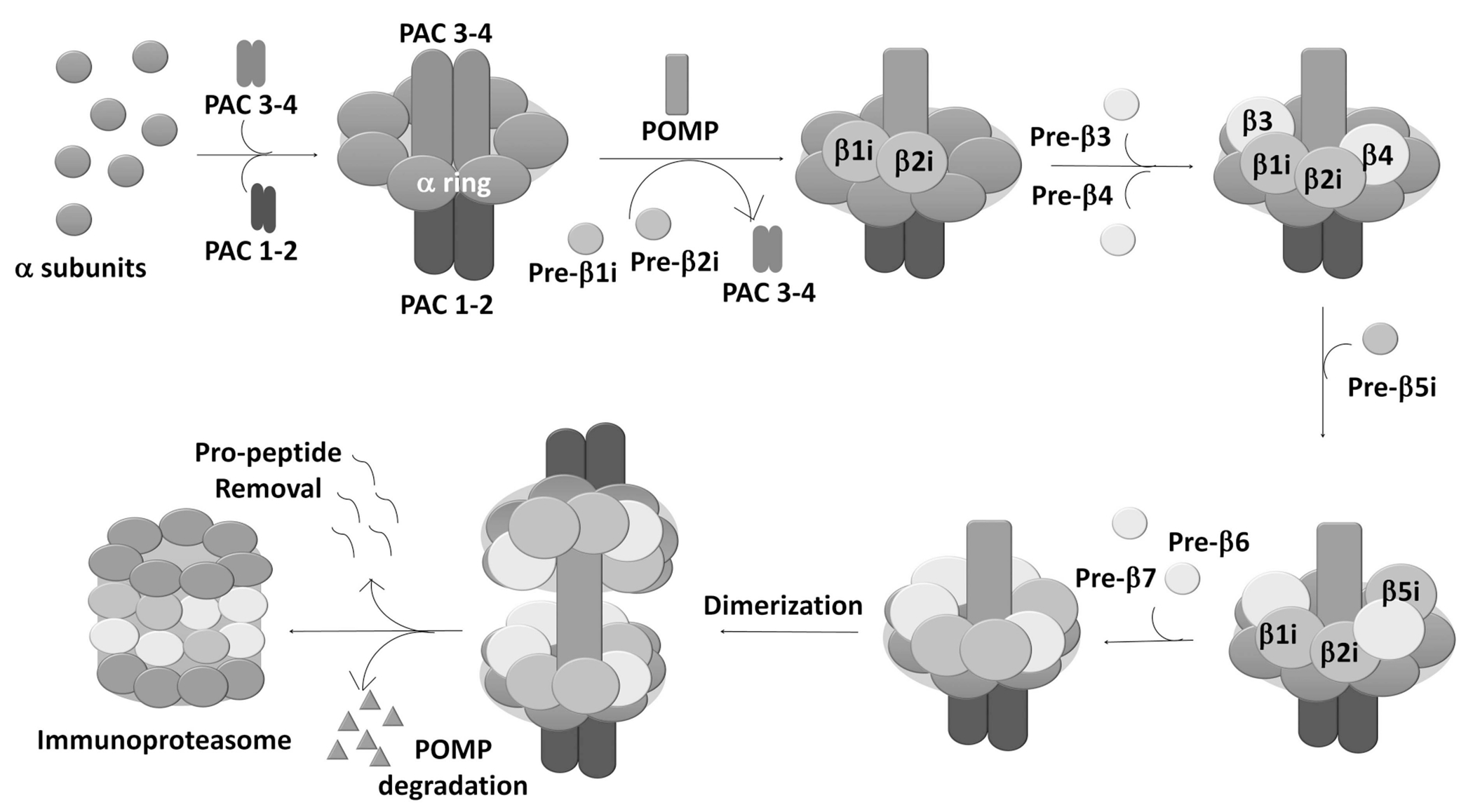
Assembly of the immunoproteasome. Immunoproteasome assembly begins with the formation of an α-ring. PAC 1 – 2 and PAC 3 – 4 assist this process. Thereupon, the pre-β1i and pre-β2i are simultaneously incorporated into the assembly through the action of POMP. The pre-β5i subunit attaches to this assembly later. Two such assemblies dimerize to form a functional proteasome. For the assembly of a fully functional immunoproteasome, POMP degradation and pro-peptide removal must occur. A full color version of this figure is available online at the *Immunology and Cell Biology* website.

The assembly of the 20S subunit of immunoproteasome is assisted by a dedicated group of chaperone proteins commonly known as the proteasome assembling chaperones (PACs). PAC 1 - PAC 2 and PAC 3 - PAC 4 are the chaperone proteins that direct α-ring assembly.^12^ The β-subunit propeptides of immunoproteasomes and constitutive proteasomes are formed simultaneously; however, their incorporation into the immunoproteasome core depends on numerous factors that include their expression and induction by infection or other triggers. Post α-ring assembly, the simultaneous incorporation of LMP2 and MECL1 occurs.^16^ The propeptides for LMP2 and MECL1, which are also known as pre-LMP2 and pre-MECL1, are cooperatively incorporated during the assembly process to form what is known as a preproteasome. A homolog of preproteasome maturation factor, Ump1, assists in this mutual incorporation and in the maturation of the immunoproteasome, and is known as proteasome maturation protein (POMP).^5^ This assembly further facilitates the incorporation of pre-LMP7 (Figure 3). Importantly, pre-LMP7 can also incorporate itself into the pre-proteasomal assembly without pre-LMP2 or pre-MECL1, which results in the formation of mixed or intermediate proteasome populations.^10^

#### Regulation.

The expression of immunoproteasomes is generally low in most cell types and increases significantly, particularly in antigenpresenting cells, upon exposure to factors including IFN-γ, IFN-α/β, TNFα, and lipopolysaccharides and oxidative stress.^5,17^ Moreover, transcription factors, such as NF-кB, Sp1, ΔP-1, CREB and Zif268/ Egr1, are known to regulate the expression of the LMP2 subunit.^5,18^ In addition, there is an evidence of cytokine-independent regulation of the immunoproteasome profile. For example, elevated levels of nitric oxide cause an increase in the immunoproteasome profile via the cGMP/cAMP pathway.^5^

#### Functions.

Immunoproteasomes exhibit both immune and non-immune functions and have a significant role in combating illnesses and infections. Although the full potential of the immunoproteasome has not yet been explored, some of the central functions of this complex that have been studied to date include the following:

#### Antigen presentation.

The principal role of immunoproteasomes in vertebrate cells is the generation of a pool of peptides with high MHC I binding affinity for antigen presentation. There is no difference between the protein degradation efficiencies or the sizes of peptides formed by standard proteasomes and immunoproteasomes;^19^ however, compared with their constitutive counterparts, immunoproteasomes have altered cleavage patterns, with preferential cleavage after non-polar subunits,^20^ thereby optimizing the quality and quantity of the generated peptides.^21^ Specifically, the cleft of the MHC class I molecule can accommodate the hydrophobic C-terminal anchor residues generated by the chymotrypsin-like activity of the LMP7 immunoproteasome subunit. Thus, it is believed that immunoproteasomes are important for generating a distinct pool of peptides. The cleavage preferences of each active subunit depend on the chemical nature of their substrate specificity pockets, which accommodate the ligand side chains of the antigenic proteins. A number of ligand docking experiments have revealed the structural differences in ligand binding and ligand cleavage patterns between constitutive proteasomes and immunoproteasomes. For example, the large substrate binding pocket of the β2 subunit endows it with a broader substrate specificity compared with MECL1 (β2i).^20^ Similarly, the β1 subunit of the constitutive proteasome induces cleavages following acidic side chains, which gives it a caspase-like activity. In contrast, the LMP2 (β1i) subunit of the immunoproteasome has a hydrophobic lining and thus preferentially cleaves branched-chain amino acids.^20,22^ Furthermore, crystal structure comparisons of mouse β5 and LMP7 (β5i) subunits have revealed that the substrate specificity pocket of β5 is smaller than that of LMP7 (β5i). Thus, in addition to chymotrypsin-like activity, β5 also exhibits elastase-like activity, whereas LMP7 exerts only chymotrypsin-like activity.^20,23^ In general, LMP2, LMP7 and β5 are believed to generate high-affinity MHC I ligands with longer retention times.^14^ Furthermore, a 2012 study tested the antigen-presenting capabilities of mice lacking all three immuno-subunits. The results showed defective MHC class I antigen presentation in the immunoproteasome-deficient mice; further supporting the idea that immunoproteasomes are crucial for MHC class I presentation.^24^

#### Maintenance of protein homeostasis and protection against oxidative stress.

The basic function of the ubiquitin proteasome system is the degradation of non-functional, misfolded proteins, and immunoproteasomes are believed to contribute to this function. Immunosubunit expression and activity are reportedly upregulated in response to stress or injury, and these findings are suggestive of the protective role of immunoproteasomes in these conditions.^25^ Furthermore, increased levels of oxidized protein products in the brains and livers of LMP2^−/−^ mice and the increased sensitivity of retinal epithelial cells obtained from double knockout LMP7^−/−^/MECL1^−/−^ mice indicate that immunoproteasomes might have a role in alleviating oxidative damage.^25,26^ However, there are contradictory reports regarding this function. Seifert *et al*.^27^ reported increased production of IFNγ in human and murine cell lines that led to increase in the expression of immune subunits. Assessments of proteasome activity in response to IFNγ have revealed a transient decrease and a subsequent increase in the proteasomal chymotrypsin-like activity after 24 h. This pattern correlates with the time required for the formation of immunoproteasomes as determined by the LMP2 incorporation experiments performed in this study. Moreover, the capacity for protein degradation was assessed by analyzing defective ribosomal products in mice deficient in one or more immunoproteasome subunits, and mice having functional immunoproteasomes were found to be more efficient in the clearance of polyubiquitinated proteins. Together,these findings led this group to conclude that immunoproteasomes are more efficient at maintaining protein homeostasis than are standard proteasomes.^27^ In another study, no significant differences were reported in terms of the degradation of ubiquitinated proteins by immunoproteasomes and standard proteasomes under identical experimental conditions.^28^ These reports clearly suggest that substantial efforts are still needed to provide confirmatory evidence regarding the role of immunoproteasomes in the maintenance of protein homeostasis.

Upon testing the role of the ubiquitin proteasome system in the maintenance of health and longevity in the naked mole rat, Rodriguez *et al*.^29^ unveiled an important facet of immunoproteasome function. In this study, the immunoproteasomes of naked mole rats were found to exhibit higher rates of trypsin- and chymotrypsin-like activities relative to those of normal mice. Naked mole rats are the longest-living rodents and maintain robust health over most of their lifespan. This characteristic led to the hypothesis that the ubiquitin proteasome system might have an integral role in the maintenance of protein homeostasis in these animals. However, no differences were found in comparisons of the proteasomes and proteasome variant profiles of naked mole rats with those of normal mice. Nonetheless, the catalytic activity of immunoproteasomes was increased in the naked mole rats compared with the normal mice. Thus, this study suggested that naked mole rats have efficient protein degradation machinery that maintains protein homeostasis and can mainly be attributed to increased immunoproteasome activity.^29^ Nevertheless, this study raises some new questions regarding the function of immunoproteasomes including whether or not these results translate to humans and lead to increased longevity and improved health.

#### Cytokine production.

Under normal conditions, an immuno- proteasome-mediated regulatory mechanism operates to lower cytokine to create a balance between destructive inflammation and effective responses to infection.^5^ The role of the immunoproteasome in these events can effectively be studied via a rare condition, Nakajo - Nishimura syndrome, which has been reported only in the Japanese population and is characterized by nodular erythema, lipomuscular atrophy and hyper-γ-globulinemia. The main cause of this disorder is the defective incorporation of the LMP7 subunit during immunoproteasome assembly, which leads to increased levels of IL-6- and IFN-γ-inducible protein-10 in the patients’ sera.^30^ In contrast, dendritic cells derived from the bone marrow of LMP2^−/−^ mice exhibit considerable reductions in the production of IFN-α, interleukin (IL)-1β, IL-6 and TNF-α in response to influenza infection compared with cells from wild-type mice. This reduction in cytokine production observed in immunoproteasome-deficient cells has been attributed to compromised NF-κB signaling, which further suggests the involvement of immunoproteasomes in cytokine production.^31^

#### T-cell proliferation.

Studies have demonstrated the role of immunoproteasomes in T-cell differentiation, survival and proliferation.^31,32^ A study conducted to test the effect of immunoproteasomes on viral antigen presentation revealed that immunoproteasomes not only affect the antigenic determinants produced for T-cell recruitment but also influence the T-cell repertoire. CD8^+^ T-cell counts have been found to be reduced in LMP2^−/−^ mice compared with wild-type (C56Bl/6) controls. Furthermore, the adoptive transfer of CD8^+^ T cells from LMP2^−/−^ to wild-type mice also results in reduced immune responses against antigenic determinants produced by both standard and immunoproteasomes. These results suggest that immunoproteasomes enhance the ability of CD8^+^ T cells to respond to foreign antigens.^33^ Similarly, Basler *et al*.^34^ found that the loss of the MECL1-inducible subunit results in a marked reduction in the number of cytotoxic T cells in response to lymphocytic choriomeningitis virus infection. Another study reported hyper-proliferation of CD4^+^ and CD8^+^ T cells and increased cell cycling of the T cells of double knockout C57BL/6 background mice lacking two immuno-subunits, MECL1 and LMP7. Notably, these findings were not observed in the absence of only one subunit. These results suggest a role for the immunoproteasome in T-cell proliferation and survival.^35^ Another report demonstrated that the inhibition of LMP7 leads to the reduced differentiation of naive CD4 T cells into Th17 cells. Interestingly, PR-957 (a LMP7 inhibitor) blocks endotoxin-stimulated T cell-mediated production of IFN-γ. These findings further suggest that LMP7 regulates the production of pro-inflammatory cytokines and the polarization of T cells.^31,32^

#### Maintenance of pluripotency.

Recently, it was determined that immunoproteasomes have a role in the maintenance of pluripotency in human embryonic stem cells. Indeed, the LMP7 gene has been found to be amongst the top ten molecular signatures of human embryonic stem cells. Upon the embryoid body-mediated differentiation of human embryonic stem cells, the loss of LMP2 and LMP7 expression at both the RNA and protein levels within 16 days of differentiation has been observed.^36^ Similarly, reports show that use of the immunoproteasome-specific inhibitors UK101 and PK257 results in decreased efficiency of cellular reprogramming and self-renewal, and increased expression of somatic markers, including FGF5 and GATA4 in human embryonic stem cells.^37^ These observations indicate the critical requirement for the immunoproteasome during the induction of pluripotency in somatic cells.

## ROLE OF THE IMMUNOPROTEASOME IN DISEASE PATHOLOGIES

### Cancers

The variation in the expression of immunoproteasomes has been attributed to the occurrence of different types of cancers. Interestingly, on one hand, the upregulation of immunoproteasome expression has been reported in prostate cancer, multiple myeloma and lung cancer, but on the other hand, the downregulation of immunoproteasome expression has been reported in cancers of the colon,^38^ kidney, skin, neck, head and esophagus.^6^ For example, abnormal MHC class I expression and the loss of antigen processing are attributes of malignant cells in human and murine models that are associated with the occurrence and progression of melanoma, breast and colorectal cancers. In this regard, 9 of 10 micro-dissected human colorectal tumor tissues were observed to exhibit decreased expression of LMP7, which correlated with the reduced expression of MHC class I antigens. It has been suggested that LMP7 is responsible for altered substrate specificity that results in the production of hydrophobic peptides. Hence, the reduced expression of the LMP7 subunit decreases the presentation of antigenic peptides by HLA molecules to CD8^+^ T cells, which results in tumor growth.^38^

**Figure 4:**
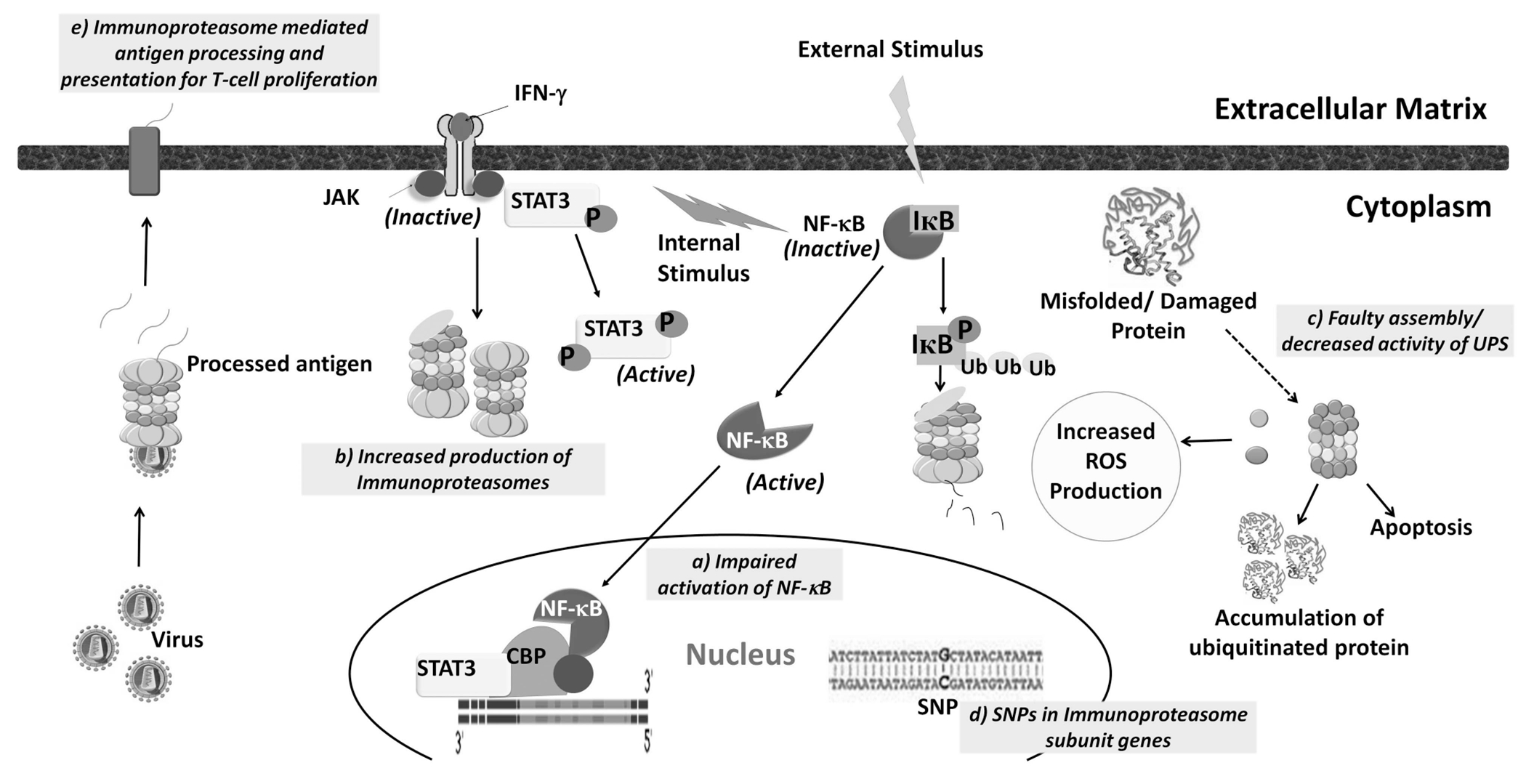
Roles of immunoproteasomes in various ailments. (**a**) Prolonged activation of the NF-κB signaling pathway via degradation of its cytoplasmic inhibitor IκB is considered an underlying cause of various cancers. Immunoproteasome inhibitors have been shown to reverse cancer growth in *in vitro*, as well as *in vivo* models. (**b**) Inflammatory diseases and microbial infections exhibit IFN-γ-mediated production of immunoproteasomes; however, overproduction of immunoproteasomes leads to heightened inflammatory responses in tissues. (**c**) In some disease pathologies, immunoproteasomes are thought to be involved in endoplasmic reticulum (ER)-related and oxidative stress responses that lead to imbalances in proteostasis, reactive oxygen species (ROS) production and apoptosis in affected lung tissues. (**d**) The presence of single-nucleotide polymorphisms (SNPs) in genes that code for immunoproteasome subunits has been found to be associated with increased susceptibility to many infections in some populations. (**e**) Immunoproteasomes function in antigen processing and presentation during microbial and viral infections. However, bacterial pathogens have been shown to hijack the host’s immunoproteasome system and thus prevent the degradation of misfolded proteins, which results in bacterial growth. A full color version of this figure is available online at the *Immunology and Cell Biology* website.

Because of its multifaceted role in several pathological conditions, the NF-κB signaling pathway is a favored target of the ubiquitin proteasome machinery. However, degradation of IκB, an inhibitor of NF-κB, by the immunoproteasome remains controversial. A study published in 1999 reported impaired activation of NF-κB in macrophages and fibroblasts from the lungs and spleens of 6-week-old LMP2^−/−^ mice^39^ and suggested that inhibition of the immunoproteasome could suppress NF-κB-mediated inflammation (Figure 4a). Moreover, bortezomib, an FDA-approved drug for multiple myeloma that is also being tested for the treatment of lung cancer, has been shown to target the NF-κB signaling pathway. It has been reported that bortezomib blocks the degradation of IκB, thereby preventing the activation of NF-κB and, in turn, NF-κB-mediated inflammatory events.^40^ However, a recent study reported that specific inhibitors of the LMP2 and LMP7 subunits had no significant effect on NF-κB signaling in lung adenocarcinoma cell lines (H23 and Panc-1). This same study reported suppressed IκB phosphorylation and subsequent inactivation of NF-κB signaling in response to the broad-spectrum proteasome inhibitor epoxomicin,^41^ which suggests that the proteasomes and not the immunoproteasomes have a role in NF-κB activation. These conflicting results may be attributed to the employed cell types and stimuli. In addition, the possibility of the involvement of alternate or mixed immunoproteasomes in the regulation of NF-κB signaling cannot be ruled out.

### Inflammatory disorders

Numerous studies have demonstrated elevated levels of immunoproteasomes in a variety of autoinflammatory and autoimmune diseases including ulcerative colitis,^42^ inflammatory bowel disease, hepatitis and Crohn’s disease.^6^ These studies provide us with a better understanding of the role of immunoproteasomes on our health and wellbeing. While reviewing the pathophysiology of inflammatory bowel disease, Fitzpatrick^43^ observed elevated levels of the LMP2 subunit in patients with inflammatory bowel disease, especially in cases of Crohn’s disease. Studies with dextran sodium sulfate -induced colitis have revealed an amelioration of disease symptoms in mice deficient in one of the immunoproteasome subunits. Furthermore, these mouse models exhibit decreased activation of NF-κB signaling and diminished expansions of Th1 and T17 cells, and these effects were thought to be consequences of normal immunoproteasome populations in these mice.

Volkov *et al*.^9^ observed significant declines in the recruitment of inflammatory cells, cytokine productions and the T helper cell populations in the bronchoalveolar lavage fluid of LMP7 knockout mice along with a reduced asthmatic phenotype compared with their littermate controls. In addition to highlighting the importance of a single inducible immunoproteasome subunit (LMP7 in this case) as a therapeutic target in asthma, these findings indicate that the roles of other inducible subunits (that is, LMP2 and MECL1) might also be important and require further investigation. It is also important to note that the LMP7 subunit can be incorporated into the proteasomal assembly independently of LMP2 or MECL1, thus giving rise to a mixed proteasome population (Figure 2). Further studies are necessary to determine the role of mixed immunoproteasomes in asthma.

Another study demonstrated an increase in the expression of the LMP2 and LMP7 subunits and elevated proteasomal enzyme activity in the bronchoalveolar lavage fluids of patients with acute respiratory distress syndrome, which suggests the involvement of immunoprotea- somes in the pathophysiology of this disease (Figure 4b).^44^ Furthermore, intriguing results were obtained in a study that compared the immunoproteasome composition and enzyme activity in lung tissues between patients with idiopathic pulmonary fibrosis, sarcoidosis and chronic obstructive pulmonary disorder (COPD). This study revealed enhanced expression and activity of the 20S proteasomal subunit in the lung tissues of patients with idiopathic pulmonary fibrosis and sarcoidosis in addition to an unaltered expression profile with reduced proteasomal enzyme activity in the lung tissue lysates from the COPD patients (Figure 4c). Considerable heterogeneity was observed regarding the subunit distributions among the specimens and was attributed to variations in the exposure to inhaled microbes and foreign particles in the subjects’ lifetimes, which made the comparison of clinical data extremely complex. The major downside of this study was the sample size, that is, only 5 – 7 individuals with each disease condition were included, which makes it difficult to draw definitive conclusions. Furthermore, the immunoproteasome populations were assessed in crude lung extracts that contained both pulmonary cells and cells from the peripheral milieu, making it difficult to determine the origin of the immunoproteasome population.^45^

A study conducted in 2011 showed an accumulation of ubiquitylated proteins in the lung tissues of COPD patients, thus indicating aggregation of misfolded and damaged proteins, likely owing to the impaired functioning of the immune-proteasomal machinery.^46^ However, a more recent study compared the immunoproteasome profiles of non-smokers, ex-smokers and COPD patients with immunoblotting and found no significant changes in the expression profiles of LMP2 or LMP7 among the COPD patients and normal donors.^47^ Such observations suggest the possibility of varying immunoproteasome-mediated responses to different environmental triggers.^46^ In addition, duration of exposure, type of inhaled particles and past infections are all likely to affect patient proteasomal population profiles and must be incorporated during data analysis in future studies.

### Infectious diseases

Environmental factors, such as infection, further affect the function of immunoproteasomes. In this context, one study showed that infecting immunoproteasome-deficient mice with *Listeria monocytogenes*, the causative agent of listeriosis, produces a negligible effect on the function of professional antigen-presenting cells, such as dendritic cells, macrophages and B cells. This effect translates into tissue-specific responses related to pathogen clearance and the frequency of CD8^+^ T-cell epitopes. In this context, LMP7^−/−^ mice exhibit impaired pathogen clearance in the liver but not in the spleen, which suggests immunoproteasome-mediated regulation of non-professional antigenpresenting cell functions.^48^ Ethanol-mediated suppression of the steady state protein levels of the immunoproteasome subunit LMP7 in addition to the impairment of chymotrypsin-like activity during hepatitis C virus infection have been reported.^49^ These processes lead to the accumulation of damaged and misfolded proteins in the lungs and also affect the antigen presentation process.^50^

Pang *et al*.^51^ demonstrated the importance of the immunoproteasome for antigen presentation and T-cell proliferation in response to influenza virus infection. This group employed the PR8 influenza A virus to infect wild-type, LMP2^−/−^,MECL1^−/−^ and MECL1^−/−^ LMP7^−/−^(C57BL/6) mice and assessed the changes in antigen presentation and CD8^+^ T-cell repertoires. The authors observed that mice deficient in one or more inducible immunoproteasome subunits switched to a subdominant status (that is, a decreased immune response) and displayed a defective repertoire of antigenic peptides compared with wild-type mice. These results indicated the important role of proteasome variants in the maintenance of immunodominance (the property of the antigenic determinant that enabled them to elicit a major immune response) of influenza-infected mice. Of note, loss of individual subunits resulted in varied patterns of changes in antigen presentation that highlighted the distinct functional roles of each subunit in antigen processing and presentation (Figure 4e). Interestingly, while studying the effect of the immunoproteasome during influenza A virus infection, Hensley *et al*.^52^ revealed that B cells purified from the spleens of C57BL/6 mice express immunoproteasomes. However, LMP2 gene-deficient mice exhibit a reduced number of B cells and a mixed population of mixed proteasomes in their lysates. The loss of the LMP2 gene in mice further results in a decreased survival ability of lymphocytes and altered NF-κB activity, thus demonstrating a crucial function of the immunoproteasome in immune cell function during infection. Nevertheless, it is difficult to deduce from such studies whether the observed results were due to the loss of LMP2 catalytic activity or faulty immunoproteasome assembly because LMP2 initiates immunoproteasome assembly.^19^ Therefore, extensive studies are needed to further delineate these possibilities.

Similar to viral infections, increases in the immunoproteasome profile have also been reported in response to fungal infections. Barton *et al*.^53^ demonstrated that the expression of LMP2, LMP7 and MECL1, as well as their regulator, PA28α/β, increases following in vivo infection with Histoplasma capsulatum, the causative agent of histoplasmosis. Furthermore, IFN-γ production has been found to directly affect the advancement of histoplasmosis infection. Specifically, IFN-γ-deficient mice exhibited no basal expression of immunoproteasome subunits (specifically LMP2) and exhibited increased histoplasmosis infection in a C57BL/6 model.

### Neurological disorders

Diseases of the central nervous system are frequently associated with imbalances between proteolysis and protein synthesis that make study of the ubiquitin proteasome system central to the understanding disease pathology. Studies performed on animal models of many neurodegenerative diseases, including Huntington’s disease, Alzheimer’s disease and amyotrophic lateral sclerosis, have revealed elevated levels of the LMP2 and LMP7 immunoproteasome subunits.^6^ In addition, while studying the disease pathophysiology of multiple sclerosis (MS), Mishto *et al*.^54^ found accumulations of immunoproteasomes and the 11S regulator in MS-affected plaques and brain areas. The expression of these genes was found to be elevated in neurons, oligodendrocytes, macrophages, endothelial cells and lymphocytes. Interestingly, a gender-based genetic study of 1262 MS patients and 845 controls from Italy revealed a decreased risk of disease occurrence among female subjects carrying the 60H variant of the LMP2 codon that indicated a gender-based disease susceptibility. *In vitro* analysis of this phenomenon using a lymphoblastoid cell line revealed that immunoproteasomes with the LMP2 60H allele produce a reduced amount of the HLA-A*0201-restricted immunodominant epitope MBP_111 – 119_. Altered production of the MBP epitope might result in attenuation of MBP-reactive CD8^+^ T-cell cytotoxicity, restraint on the clonal expansion of CD8^+^ T cells and altered blood – brain barrier crossing of immunogenic cells, which thereby contributes to reduced disease progression. Therefore, this study not only demonstrated the role of immunoproteasomes but also suggested the gender dependency of the genetic factors associated with MS. Considering the harsh side effects of bortezomib, which is currently used as a therapeutic agent for MS, the immunoproteasome is an attractive target for the potential development of novel therapies.

### Host – pathogen mutations/single-nucleotide polymorphisms

The LMP2 and LMP7 genes of the immunoproteasome machinery are highly polymorphic and map within the class II region of the MHC.^55,56^ Thus, it is not surprising that polymorphisms in these genes might lead to various genetic conditions including, joint contractures, muscle atrophy, microcytic anemia and panniculitis-induced lipodystrophy syndrome and Nakajo - Nishimura syndrome. Mutations of the LMP7 gene are associated with the occurrence of panniculitis-induced lipodystrophy syndrome in people from Portugal and Mexico. A homozygosity mapping report based on patients suffering from this disease identified a homozygous region spanning 2.4 Mb on chromosome 6p21 that was shared by all affected individuals. In addition, a missense mutation, c.224C>T (pThr75Met), in the LMP7 subunit gene was identified in all the individuals from both the lineages. Further analysis revealed that the substitution of methionine for threonine at position 75 significantly disrupted the tertiary structure of LMP7 and thereby resulted in a reduction in the chymotrypsin activity of the protein and a reduction in disease symptoms.^57^ Similarly, a homozygous mutation in the LMP7 gene can cause Nakajo - Nishimura syndrome, a distinct inherited inflammatory and wasting disease originally reported in Japan.^58^ Alarcon *et al*.^59^ evaluated LMP gene polymorphisms as markers of spondyloarthritis in the Mexican population. Genotype analysis revealed an increased frequency of the LMP2 R/R genotype among the patients with spondyloarthritis as compared with controls, and thus the LPM2 polymorphism was linked to increased susceptibility to spondyloarthritis.

In some cases, minor variations of the genes of the host immunoproteasome system increase susceptibility to certain types of infection. For example, a gene variant at the LMP7 codon position 145 (Gln–Lys) is associated with increased susceptibility to intestinal *Mycobacterium tuberculosis* infection in the Chinese population; however, this mutation has no effect on the susceptibility to pulmonary tuberculosis.^60^ Similarly, a study conducted on a Mexican population found that a gene polymorphism at amino acid position 49 (Gln/Lys) in the second exon of the LMP7 gene is associated with increased susceptibility to hypersensitivity pneumonitis, but the same polymorphism has no effect on disease susceptibility in other populations.^61^ Therefore, it is difficult to draw definitive conclusions from these results because the associated studies did not take into account environmental exposure to causative pathogens. Moreover, the lack of disease occurrence in populations with the same mutations could simply be the result of their lack of exposure to the virulent form of the pathogen. Although notion does not completely undermine the effects of LMP polymorphisms on the occurrence of various diseases, further research is required to generate a greater understanding of this phenomenon and to enable better screening for and management of clinical conditions (Figure 4d).

## FUTURE PROSPECTS

### Immunoproteasomes as drug targets

Constitutive proteasomes have been successfully targeted since 2003, when the FDA-approved bortezomib acted as a first-line treatment for multiple myelomas. Bortezomib is a boronic acid analog that inhibits the chymotrypsin-like activity of both proteasomes and immuno- proteasomes.^62^ Given the successful use of bortezomib for multiple myelomas, its effectiveness against various types of solid tumors has been investigated.^40^ For example, a pre-clinical study showed that bortezomib-mediated inhibition of NF-κB increases the sensitivity of NSCLC cells to gemcitabine-induced apoptosis.^63^ In addition, phase I and II studies have demonstrated the potential of combination therapy with bortezomib and available chemotherapeutic agents for the treatment of NSCLC, and details of these studies are available at www.clinicaltrials.gov.^40^ However, side effects associated with borte-zomib therapy have encouraged development of subunit-specific inhibitors. Consequently, many immunoproteasome-specific inhibitors, including PR-924, ONX0914, YU102, IPSI001 and UK101, have been developed and are being tested for their efficacy in various *in vitro* and *in vivo* models.^6^ Although these studies are in their preliminary stages, promising outcomes are expected in the near future.

### Immunoproteasomes as biomarkers

The roles of immunoproteasomes in various disease conditions suggest that they may be useful biomarkers. For example, defective LMP2 expression has been identified as one of the risk factors for uterine leiomyosarcoma and has further been suggested to act not only as a potential biomarker but also as a drug target for this condition.^64^ Similarly, another study showed that the C-terminal fragment of the 11S immunoproteasome activator (PA28) is specifically found in biopsies from ovarian cancer tissues and not in benign tumors. Therefore, it has been proposed that this peptide could be employed as a marker to identify early-stage ovarian cancer while it is still treatable.^65^ Another report further suggested the possibility of employing LMP2 as a potential biomarker for human tumors.^66^ In addition, as mentioned above, there are reports of single-nucleotide polymorphisms in immunoproteasomal subunit genes that are associated with changes in disease risk or incidence in various populations.^57,60,61^ These may serve as biomarkers for these conditions and aid in genetic screening of susceptible groups.

### Immunoproteasomes for vaccine development

Immunoproteasomes have pivotal roles in antigen presentation and the CD8^+^ T cell-mediated response to viral infections. These roles are underscored by the finding that following lymphocytic choriomeningitis virus infection, the standard proteasomes found in the liver are almost completely replaced by immunoproteasomes within 7 days. Moreover, Khan et al.^67^ reported that, during lymphocytic choriomeningitis virus infection, the CD8^+^ T-cell response is primarily directed toward immunoproteasome-dependent T-cell epitopes. This finding suggests that the peptides used in vaccines against lymphocytic choriomeningitis virus infection should possess epitopes reflective of those processed by the immunoproteasome to produce a complete T-cell repertoire against the viral antigen.^19^ To this end, a computational model was recently developed to predict proteasomal and immunoproteasomal cleavage sites in peptides. Excitingly, the cleavage sites identified were relevant CD8^+^ T-cell epitopes, and this model may thus be useful in the design of epitope-based vaccines to elicit T-cell responses.^68^ Further research exploring the possibility of targeting the immunoproteasome machinery for vaccine development is needed in the future.

## CONCLUSION

Considering the role of immunoproteasomes in disease pathogenesis, further study of this complex will likely reveal unique mechanisms underlying various ailments. This knowledge will certainly prove useful in the design of future therapeutic interventions; however, forthcoming research in this regard needs to be more focused. To date, studies have assessed the involvement of the total proteasome population of a cell to determine the roles of these complexes in lung diseases.^9,45^ With the advent of novel techniques that can target and purify immunoproteasomes, improved assessment of their role in disease conditions will be possible.^69^ Furthermore, it will be important to differentiate between the immunoproteasome and mixed proteasome populations in order to attain more conclusive results.

## Figures and Tables

**Table 1:** Differences between constitutive proteasomes and immunoproteasomes

Characteristic	Constitutive proteasomes	Immunoproteasomes	Reference
Occurrence	Conserved in all eukaryotes	Conserved in jawed vertebrates	70
β-Subunits	β_1_ (PSMB6, Y, δ) β_2_ (PSMB7, Z, MC14) β_5_ (PSMB5, X,MB1,ε)	β_1i_ (PSMB9, LMP2) β_2i_ (PSMB10, MECL1) β_5i_ (PSMB8, LMP7)	31
Assembly	Slower (~ 82 min)	Faster ( ~21 min)	1
Nature of peptide degradation	ATP dependent	Both ATP dependent and ATP independent	13
Protease activity	β_1_: caspase-like activity β_2_: trypsin-like activity β_5_: chymotrypsin-like activity	β_1i_: ↓caspase-like activity β_2i_: ↑trypsin-like activity β_5i_: ↑chymotrypsin-like activity	6
Chief role	Protein homeostasis and degradation	Produce peptides for antigen presentation.	6
Half life	Longer (~133 h)	Shorter ( ~ 27 h)	1,31
Cleavage site	Stimulate cleavage after acidic residues	Stimulate cleavage after hydrophobic, basic and branched-chain residues	57
